# Prognostication of neurologic outcome using gray-white-matter-ratio in comatose patients after cardiac arrest

**DOI:** 10.1186/s12883-021-02480-6

**Published:** 2021-11-22

**Authors:** Konrad Kirsch, Stefan Heymel, Albrecht Günther, Kathleen Vahl, Thorsten Schmidt, Dominik Michalski, Michael Fritzenwanger, Paul Christian Schulze, Rüdiger Pfeifer

**Affiliations:** 1 Department of Internal Medicine I, Jena University Hospital, Am Klinikum 1, 07747 Jena, Germany; 2 Department of Internal Medicine I, Division of Medical Intensive Care, Jena University Hospital, Am Klinikum 1, 07747 Jena, Germany; 3 Department of Neurology, Jena University Hospital, Am Klinikum 1, 07747 Jena, Germany; 4 Department of Radiology, Interventional Radiology and Neuroradiology, Klinikum Altenburger Land, Am Waldessaum 10, 04600 Altenburg, Germany; 5 Department of Diagnostic and Interventional Neuroradiology, HELIOS Klinikum Wuppertal, Heusnerstraße 40, 42283 Wuppertal, Germany; 6 Department of Neurology, University Hospital Leipzig, Liebigstraße 20, 04103 Leipzig, Germany

**Keywords:** Heart arrest, Cardiopulmonary resuscitation, Hypoxia-ischemia, brain, Neuroimaging, Coma, Prognosis, Retrospective studies, Predictive value of tests

## Abstract

**Background:**

This study aimed to assess the prognostic value regarding neurologic outcome of CT neuroimaging based Gray-White-Matter-Ratio measurement in patients after resuscitation from cardiac arrest.

**Methods:**

We retrospectively evaluated CT neuroimaging studies of 91 comatose patients resuscitated from cardiac arrest and 46 non-comatose controls. We tested the diagnostic performance of Gray-White-Matter-Ratio compared with established morphologic signs of hypoxic-ischaemic brain injury, e. g. loss of distinction between gray and white matter, and laboratory parameters, i. e. neuron-specific enolase, for the prediction of poor neurologic outcomes after resuscitated cardiac arrest. Primary endpoint was neurologic function assessed with cerebral performance category score 30 days after the index event.

**Results:**

Gray-White-Matter-Ratio showed encouraging interobserver variability (ICC 0.670 [95% CI: 0.592–0.741] compared to assessment of established morphologic signs of hypoxic-ischaemic brain injury (Fleiss kappa 0.389 [95% CI: 0.320–0.457]) in CT neuroimaging studies. It correlated with cerebral performance category score with lower Gray-White-Matter-Ratios associated with unfavourable neurologic outcomes. A cut-off of 1.17 derived from the control population predicted unfavourable neurologic outcomes in adult survivors of cardiac arrest with 100% specificity, 50.3% sensitivity, 100% positive predictive value, and 39.3% negative predictive value. Gray-White-Matter-Ratio prognostic power depended on the time interval between circulatory arrest and CT imaging, with increasing sensitivity the later the image acquisition was executed.

**Conclusions:**

A reduced Gray-White-Matter-Ratio is a highly specific prognostic marker of poor neurologic outcomes early after resuscitation from cardiac arrest. Sensitivity seems to be dependent on the time interval between circulatory arrest and image acquisition, with limited value within the first 12 h.

## Background

Sudden cardiac arrest is among the leading causes of death in industrialized countries, incidence of out of hospital cardiac arrest (OHCA) with attempted cardiopulmonary resuscitation (CPR) is estimated to be as high as approximately 50 to 60 per 100.000 population per year [1, 2]. Hypoxic-ischaemic brain injury is considered to be the main contributor to mortality in patients after resuscitation from cardiac arrest [3]. Moreover, it accounts for at least notable morbidity and cognitive impairment in survivors of cardiac arrest [4, 5]. Early prognostication of neurologic outcome is therefore crucial in order to identify patients, in whom an aggressive treatment of post-resuscitation syndrome and the underlying disease will not prove beneficial or may contradict with the patient’s presumed will concerning the extent of treatment in consideration of the achievable functional status.

Various methods of prognostication are hampered by the effects of therapeutic interventions, primarily hypothermia in the course of targeted temperature management, sedative and neuromuscular blocking agents, and their potentially altered metabolism in the setting of post-resuscitation syndrome. International guidelines recommend prognostication of outcome not before 72 h after cardiac arrest or regaining normothermia in patients in whom targeted temperature management with hypothermia was applied, respectively [6, 7]. Validated predictors of a poor neurologic outcome after cardiac arrest with best diagnostic accuracy, i. e. a false positive rate near 0% with a narrow 95% confidence interval, are absent pupillary light reflexes at 72 to 108 h after cardiac arrest, and bilateral absence of the N20 waveform of somatosensory evoked potentials (SSEPs) 24 to 72 h after cardiac arrest or rewarming [7]. Electroencephalography, biomarkers neuron-specific enolase (NSE) and S-100B, and brain imaging with either computed tomography (CT) or magnetic resonance imaging (MRI) may be used as adjunctive tests in combination with other methods. It is therefore prudent to utilise more than one prognostic test in order to avoid false positive test results and thus erroneously recommend withdrawal of life-sustaining treatments (WLST) in patients with possibly favourable outcomes.

Concerning imaging-based parameters, CT is feasible in intensive care patients, widely available, and rapidly performed. In CT neuroimaging decreased attenuation of gray matter, subsequent loss of distinction of the junction between gray and white matter, and diminished cerebrospinal fluid spaces are the morphologic features of generalised cerebral oedema [8], the pathologic hallmark of severe hypoxic-ischaemic brain injury. Recent studies suggested that calculation of gray-white matter ratio (GWR), i. e. the quotient of tissue x-ray attenuation in distinct regions of interest (ROIs) in gray and white matter, could reduce interobserver variability and therefore enhance diagnostic accuracy in predicting a poor neurologic outcome. However, to date there is no consensus regarding absolute thresholds for GWR and the timing of CT acquisition, as well as the impact of targeted temperature management with mild hypothermia. Concerning the timing of neuroimaging the practical benefit of an immediate post-arrest CT scan, capable of ruling out cerebral causes of cardiac arrest as well as possible head trauma, seemingly has to be weighed against the prognostic potential, as many pathologic effects of the interruption of cerebral perfusion appear with temporal delay [9, 10]. Therefore, this study aims to investigate the prognostic value of CT imaging with a special focus on GWR measurement within the first three days after circulatory arrest.

## Methods

### Study setup

In this retrospective observational investigation, CT neuroimaging studies of patients resuscitated from cardiac arrest were assessed concerning morphologic features and GWR by a set of two independent neuroradiologists and two neurologists. Furthermore, demographic, clinical, and laboratory data were obtained. The study protocol was approved by the institution’s ethics committee (approval ID 3672–01/13).

### Patient selection

This study was conducted at the University Hospital Jena, which serves as a tertiary referral centre for a population of approximately 1.5 million people. At our institution all patients admitted to the medical intensive care unit (ICU) for post-reanimation treatment are entered into a CPR registry. Using this registry we identified 92 patients admitted to the medical ICU between March 2004 and September 2012 after successful resuscitation from cardiac arrest, who remained comatose after return of spontaneous circulation (ROSC) and underwent neuroimaging with non-contrast enhanced computed tomography (CT) within 3 days of admission. In addition to this patient cohort we included 46 age-matched patients, who underwent cranial CT imaging in the Emergency Department of our institution for evaluation of head injury after syncope or falls, as a control population. In this control population GWR was calculated and its distribution assessed, in order to a priori define a lower limit of normal, whose prognostic usefulness would be tested in the group of comatose patients.

### Clinical parameters

Each patient’s medical record in the hospital information system and the ICU-specific patient data management system was reviewed and relevant demographic, clinical, and laboratory data obtained.

### Computed tomography

The indication, choice of modality and timing of neuroimaging were made at discretion of the treating physicians. CT scans were obtained on a GE LightSpeed 16 scanner until October 2007, and on a GE LightSpeed VCT scanner thereafter (both devices manufactured by GE Healthcare, Little Chalfont, UK) using imaging protocols established in routine clinical care. The CT images relevant for our study were exported in DICOM format from the Picture Archiving and Communication System (PACS) of our institution and reviewed by four independent investigators (two neuroradiologists and two neurologists), blinded for history, group assignment and outcome. The workstation and software used were left at the investigator’s discretion. The evaluation protocol required assessment of morphologic features of hypoxic-ischaemic brain injury, i. e. diffuse cerebral oedema, effacement of the junction between gray and white matter, and diffuse hypodensity of cerebral parenchyma. Furthermore, readers measured radiodensity of the caudate nucleus and the posterior limb of the internal capsule bilaterally. Findings of Gentsch et al. [11] indicated noninferiority of a simplified method of obtaining GWR by measurement of four ROIs as compared with the standard method using 16 ROIs. Unpublished data [12] demonstrated equivalent results for gray matter attenuation with ROIs placed at the putamen or caudate nucleus, respectively. Radiodensity was expressed as mean attenuation in Hounsfield units and GWR subsequently calculated as the ratio of radiodensity of the caudate nucleus and the internal capsule.

### Outcomes

Neurological outcome was determined by review of the electronical patient record using the cerebral performance category (CPC) score [13, 14] at day 30 or hospital discharge, whichever occurred first. Favourable outcome was defined as good recovery (CPC 1), moderate disability (CPC 2), or severe disability (CPC 3), whereas poor outcome comprised persistent vegetative state (CPC 4), and death (CPC 5).

### Statistical analysis

Statistical analyses were performed using SPSS Statistics version 24.0 (IBM Corporation, Armonk NY, USA) and MedCalc Statistical Software version 19.1.7 (MedCalc Software Ltd., Ostend, Belgium). Categorical variables were expressed as numbers of patients (percentages) and continuous variables were expressed as median (IQR) or mean (95% confidence interval). Comparison of continuous variables was carried out using t-test or Welch test if normally distributed, or Mann-Whitney-U-Test if non-normally distributed, as assessed with Shapiro-Wilk normality test. In case of comparison of more than two groups, Kruskal-Wallis-Test was used. Categorical variables were tested with *Χ*^*2*^ tests. Different ROC curves were compared with DeLong’s method. Tests were two-sided and a *p*-value < 0.05 was considered as statistically significant.

## Results

### Patient demographics

One patient was underaged and therefore excluded, resulting in the analysis of 91 individuals in the patient cohort, and 46 controls. Mean age did not differ significantly between the two groups, 64.0 years vs. 65.0 years (*p* = 0.728), respectively. Regarding sex the groups were not balanced, proportion of females in the control group 52%, and in the patient cohort 25% (*p* = 0.002). 24% of patients had a favourable neurologic outcome after 30 days of admission, all-cause mortality was 64, 12% of patients remained in a persistent vegetative state (CPC 4). Baseline characteristics of patients with favourable neurologic outcome and patients with poor outcome were similar with respect to age, sex, setting of cardiac arrest, targeted temperature management with hypothermia, mechanical circulatory support, renal function, and APACHE II and SAPS II scores. Patients with a favourable outcome had significantly lower levels of lactate at admission and neuron-specific enolase at three days of admission, showed a shockable rhythm at first medical contact more frequently, and had shorter times to ROSC (Table 1).

**Table 1:** Patient demographics

	All patients	Poor Outcome	Favourable Outcome	*p*-value
Number of patients	91 (100)	69 (76)	22 (24)	
Age	63.5 (60.2–66.8)	64.9 (61.4–68.3)	61.5 (53.7–69.3)	0.263^b^
Female sex	23 (25)	16 (23)	7 (32)	0.400^a^
OHCA	57 (63)	44 (64)	13 (59)	0.223^a^
Time to ROSC (min)	27 (23–30)	28 (24–33)	19 (10–28)	**0.024**^b^
First documented rhythm shockable	32 (35)	18 (26)	14 (64)	**0.001**^a^
Mechanical circulatory support	13 (14)	9 (13)	4 (17)	0.868^a^
IABP	10 (11)	7 (10)	3 (13)	
ECMO	3 (3)	2 (3)	1 (4)	
Hypothermia 33 °C	53 (58)	42 (61)	11 (50)	0.365^a^
Lactate	7.2 (6.2–8.2)	7.9 (6.7–9.2)	5.1 (3.4–6.7)	**0.018**^b^
NSE	108.8 (84.3–133.2)	139.4 (109.8–168.9)	20.3 (14.7–25.9)	**< 0.001**^b^
eGFR	51.4 (46.2–56.7)	50.5 (45.2–55.9)	52.9 (38.1–67.7)	0.700^b^
APACHE II	29 (26–31)	28 (26–31)	29 (23–35)	0.770^a^
SAPS II	58 (54–62)	59 (55–64)	55 (45–65)	0.401^a^

Data are numbers of patients and percentages or means and 95% confidence intervals. *OHCA* Out of hospital cardiac arrest, *ROSC* Return of spontaneous circulation, *IABP* Intra-aortic balloon pump, *ECMO* Extracorporeal membrane oxygenation, *NSE* Neuron-specific enolase, *eGFR* estimated glomerular filtration rate, *APACHE II* Acute Physiology And Chronic Health Evaluation II score; *SAPS II* Simplified Acute Physiology Score. ^a^Chi-squared test, ^b^t-test

### CT findings

Assessment of all CT studies regardless of group assignment restricted to qualitative judgment relating to morphologic features of hypoxic-ischaemic brain injury and relevant differential diagnoses in patients after resuscitation from cardiac arrest showed moderate overall interobserver correlation, Fleiss kappa 0.389 (95% CI: 0.320–0.457), with marked dispersion of diagnostic accuracy for the prediction of worse neurologic outcomes; sensitivity ranged from 69.6 to 89.9%, and specificity from 46.3 to 91.2%, respectively (Table 2).

**Table 2:** Evaluation of CT morphologic features of hypoxic-ischaemic brain injury and related diagnostic accuracy regarding poor neurologic outcomes (complete study population)

	Observer A	Observer B	Observer C	Observer D	Kappa
Loss of gray and white matter distinction	58.1%	68.1%	37.0%	31.9%	0.438 (0.373–0.503)
Cerebral oedema	32.6%	60.1%	26.8%	28.3%	0.471 (0.403–0.538)
Intracranial haemorrhage	2.2%	2.9%	1.4%	0.7%	0.185 (0.116–0.254)
Ischaemia	11.5%	15.8%	4.3%	18.7%	0.224 (0.155–0.293)
Data are percentages of patients or correlation coefficients (Fleiss kappa) with 95% confidence intervals					
Sensitivity	89.9% (80.2–95.8%)	88.4% (78.4–94.9%)	69.6% (57.3–80.1%)	72.5% (60.4–82.5%)	
Specificity	52.2% (39.8–64.4%)	46.3% (34.0–58.9%)	91.2% (81.8–96.7%)	71.0% (58.8–81.3%)	
Positive predictive value	65.2% (59.2–70.9%)	62.9% (57.2–68.3%)	88.9% (78.6–94.6%)	71.4%(62.7–78.8%)	
Negative predictive Value	83.7% (71.1–91.5%)	79.5% (65.8–88.7%)	74.7% (67.2–81.0%)	72.0% (63.1–79.6%)	

Data are proportions with 95% confidence intervals

### Measurement of gray-white-matter-ratio (GWR)

**Fig. 1:**
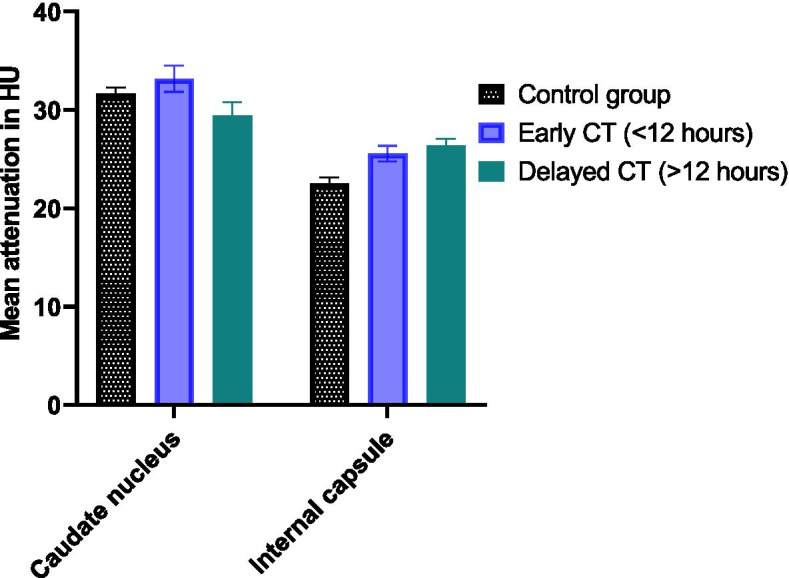
Change in attenuation with respect to CT timing

Measurement of GWR at basal ganglia showed good absolute interobserver agreement, intraclass correlation coefficient 0.670 (95% CI: 0.592–0.741). Differences in GWR were based on a decreased attenuation of gray matter, as well as an increased attenuation of white matter. Furthermore, the magnitude of this effect seemed to depend on the time interval between the circulatory arrest and acquisition of CT images (Fig. 1). In our study median time interval from admission to the ICU and CT imaging was 34 h (IQR 5–54 h). In patients with poor outcome GWR differed significantly between quartiles of time interval between admission an CT imaging, as tested with Kruskal-Wallis-Test (*p* < 0.001).

**Fig. 2:**
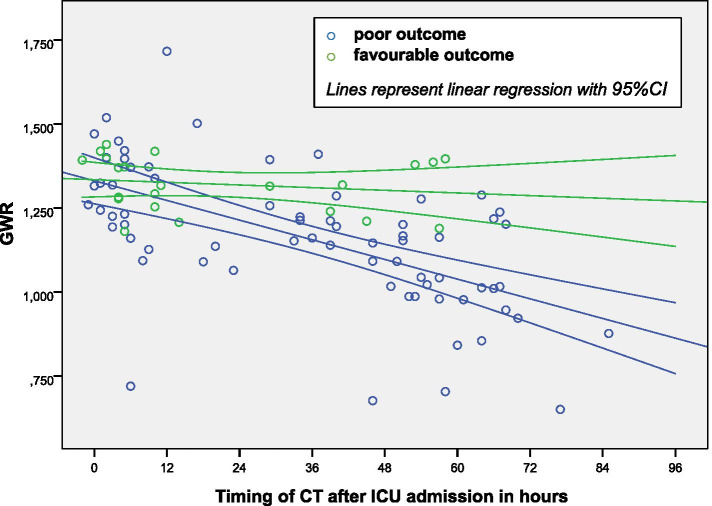
Dependency of GWR on CT timing

GWR differed significantly between patients with poor and favourable neurologic outcomes, 1.16 (95% CI: 1.11–1.21) vs. 1.32 (95% CI: 1.28–1.36; p < 0.001), respectively. As mentioned above, difference in GWR markedly increased with time interval between cardiac arrest and CT imaging, becoming significant in CT studies obtained after 12 h of admission to the ICU (Fig. 2, Table 3).

**Table 3:** GWR in selected subgroups of patients

	Poor outcome		Favourable outcome		
	n	GWR	n	GWR	*p*-value
Time interval admission to CT					
≤12 h	23 (25)	1.298 (1.216–1.380)	13 (14)	1.339 (1.292–1.386)	0.462^a^
> 12 h	46 (51)	1.091 (1.038–1.145)	9 (10)	1.293 (1.229–1.357)	**0.002**^a^
Targeted temperature management					
Mild hypothermia	42 (46)	1.133 (1.059–1.206)	11 (12)	1.304 (1.256–1.352)	**0.023**^a^
Normothermia	27 (30)	1.203 (1.147–1.259)	11 (12)	1.336 (1.274–1.397)	**0.007**^a^

Data are numbers of patients and percentages or means and 95% confidence intervals. ^a^Mann-Whitney-U-Test

Therefore, in a post hoc analysis the patient cohort was divided into two subgroups based on the timing of CT imaging: less than or equal 12 h (early CT, *n* = 36), and later than 12 h (delayed CT, *n* = 55) after admission. GWR correlated with neurologic outcome at 30 days of admission or hospital discharge, Spearman’s rank correlation coefficient 0.624 (*p* < 0.001). GWR was significantly lower in all comatose patients irrespective of timing of imaging, than in non-comatose controls, 1.20 (95% CI: 1.16–1.24) and 1.42 (95% CI: 1.39–1.45; p < 0.001), respectively. In the subgroup of patients with delayed CT imaging there was a significant difference in mean GWR with respect to neurologic outcome, 1.09 (95% CI: 1.04–1.15) for a poor outcome, and 1.29 (95% CI: 1.23–1.36, *p* = 0.002) for a favourable outcome, respectively. Patients, who survived with no neurological residues, showed no significant difference in GWR after 12 h compared to controls, 1.35 (95% CI: 1.23–1.47) vs. 1.42 (95% CI: 1.39–1.45, *p* = 0.185).

Targeted temperature management with mild hypothermia seemed to have no relevant influence on GWR, as tested with correlation ratio (Eta coefficient 0.192). Patients with poor outcome who underwent targeted temperature management with hypothermia showed no difference in GWR compared to patients in normothermia (*p* = 0.171). This also applies to patients with favourable neurologic outcome (*p* = 0.371).

### Prognostication of poor outcome

GWR in the control group was approximately normally distributed, as assessed by Shapiro-Wilk-Test (p = 0.171). Based on a mean GWR in healthy controls of 1.42 (SD 0.10) we assumed a normal range of 2.5 standard deviations around the mean, resulting in a lower limit of normal of 1.17. Applying this threshold to the subgroup of patients with delayed neuroimaging resulted in 67.4% sensitivity (95% CI: 52.0–80.5), 100% specificity (95% CI: 66.4–100.0), 100% positive predictive value (95% CI: 82.7–100.0), and 37.5% negative predictive value (95% CI: 27.4–48.8) for prediction of a poor neurologic outcome (Table 4).

**Table 4:** Diagnostic power of GWR (cutoff 1.17 for poor neurologic outcome) in different subgroups

Population	Sensitivity	Specificity	ppv	npv	AUC
*Neurologic outcome*					
All patients (*n* = 91)	50.7 (38.4–63.0)	100 (84.6–100)	100	39.3 (33.7–45.1)	0.754 (0.652–0.838)
Time interval admission to CT					
≤12 h (n = 36)	17.4 (5.0–28.8)	100 (75.3–100)	100	40.6 (36.2–45.2)	0.587 (0.411–0.748)
> 12 h (*n* = 55)	67.4 (52.0–80.5)	100 (66.4–100)	100	37.5 (28.4–47.6)	0.837 (0.713–0.923)
Targeted temperature management					
Mild hypothermia (*n* = 53)	59.5 (43.4–74.4)	100 (71.5–100)	100	39.3 (31.0–48.3)	0.798 (0.665–0.895)
Normothermia (*n* = 38)	37.0 (19.4–57.6)	100 (71.5–100)	100	39.3 (32.6–46.4)	0.685 (0.524–0.826)
*Mortality*					
All patients (n = 91)	51.7 (38.2–65.0)	84.8 (68.1–94.9)	85.7 (72.0–93.3)	50.0 (42.5–57.5)	0.683 (0.577–0.776)
Time interval admission to CT					
≤12 h (n = 36)	15.8 (3.4–39.6)	94.1 (71.3–99.9)	75.0 (25.6–96.3)	50.0 (44.3–55.7)	0.550 (0.375–0.715)
> 12 h (n = 55)	69.2 (52.4–83.0)	75.0 (47.6–92.7)	87.1 (73.8–94.2)	50.0 (36.6–63.4)	0.721 (0.584–0.834)
Targeted temperature management					
Mild hypothermia (n = 53)	60.0 (42.1–76.1)	77.8 (52.4–93.6)	84.0 (68.0–92.9)	50.0 (38.3–61.7)	0.689 (0.547–0.809)
Normothermia (n = 38)	39.1 (19.7–61.5)	93.3 (68.1–99.8)	90.0 (55.9–98.5)	50.0 (41.2–58.8)	0.662 (0.491–0.807)

Data are percentages and 95% confidence intervals. *Ppv* positive predictive value, *npv* negative predictive value, *AUC* area under the curve

ROC curve analysis in patients with delayed CT imaging showed that a GWR threshold of 1.18 predicted a poor neurologic outcome with a false positive rate of 0% (AUC 0.855, 95% CI: 0.751–0.959, *p* = 0.001). Diagnostic power of GWR for prediction of a poor neurologic outcome as measured by AUC was comparable to NSE at day 3 of ICU treatment (AUC 0.932, 95% CI: 0.831–0.982, p for comparison 0.098) and significantly better than serum lactate level on admission (AUC 0.618, 95% CI: 0.477–0.746, p for comparison 0.031). NSE levels on day 3 were available for 89 of 91 patients, and absolute cut-offs for poor outcomes were 39.4 μg/l for patients treated with TTM, and 76.1 μg/l for patients not treated with TTM, respectively. Serum lactate levels on admission were available for all patients, the absolute cut-off for poor outcomes was 13 mmol/l.

## Discussion

In the search for parameters with a prognostic value for the prediction of worse neurologic outcomes after successful resuscitation from cardiac arrest, i. e. death or persistent vegetative state, that are not influenced by sedation, we evaluated two methods based on CT neuroimaging. In contrast to most previous studies we a priori defined a cut-off for GWR derived from an age-matched non-comatose control population. Moreover, we expanded the considered time frame for CT imaging to 72 h after ROSC, on one hand in order to include as many clinically indicated imaging studies as possible, and on the other hand because most aforementioned validated predictors gain their prognostic potential only after this period.

Qualitative assessment of signs of hypoxic-ischaemic brain injury in CT imaging studies established in clinical practice yielded high sensitivity in our study. Unfortunately, this was counterbalanced by unacceptable modest specificity and considerable interobserver variability, whereas determination of GWR proved robust in this respect. GWR showed excellent interobserver agreement, even when performed by non-radiologists, and satisfactory specificity. Regarding specificity of GWR we observed a dependency of GWR on timing of CT imaging after circulatory arrest, suggesting an interval of approximately 12 h with diminished sensitivity. This presumably reflects the time course of the underlying evolution of cerebral oedema [15]. Our finding is corroborated by recent reports by Wang et al. [16] and Streitberger et al. [17], which stated a higher diagnostic accuracy of GWR when CTs were performed more than 24 h after cardiac arrest, compared to early imaging within 24 h after the initial event. Scheel et al. [18] also reported an association of lower GWR with longer delays between CPR and CT imaging, yet differences did not reach statistical significance in their study. In line with our data Yamamura et al. [19] and Lee et al. [20] did not find a difference in GWR between patients with good and poor outcomes in a cohort with CT imaging obtained within 2 h and 6 h of ROSC, respectively.

Utilising the threshold of 1.17 derived from our control population demonstrated satisfactory diagnostic accuracy in predicting poor neurologic outcomes. Only few studies have reported prespecified GWR thresholds so far [21–23], ranging from 1.10 to 1.20. The sensitivities and specificities for poor neurological outcomes found in these studies ranged from 13 to 17%, and 74 to 96%, respectively. A recent meta-analysis by Lopez Soto et al. [24] reported excellent specificity of 97% with only low to modest sensitivity ranging from 18 to 64% for GWR thresholds of 1.10 to 1.23. However, many trials included in this meta-analysis investigated CT studies acquired within 6 h after ROSC [19, 20, 25–27], used GWR cutoffs derived from post hoc analyses and exhibited considerable variation regarding quantity and localisation of the ROIs for measuring x-ray attenuation. Trials with best comparability with our study in terms of timing and ROI placement [11, 16, 18, 22] reported GWR thresholds between 1.16 and 1.18.

Concerning potential effects of the routinely applied temperature management, we did not observe an effect of mild therapeutic hypothermia on the capability of GWR to indicate an unfavourable neurologic outcome, however the number of cases in this study was insufficient to reliably exclude such effect.

### Limitations

The present study has some limitations that need to be considered. First, results of the neuroimaging studies were known to the requesting physicians and might have biased outcome through support of WLST decisions. Nevertheless, due to the retrospective design of our study GWR measurements were not available to the treating physicians and therefore had no influence on treatment decisions. Besides that, a recent neuropathology study by Endisch et al. [28] underscored the strong link between low GWR and histopathologically severe hypoxic-ischaemic encephalopathy. Second, because of its limitation to patients who remained comatose after ROSC it comprises a highly selected population with a priori limited prognosis. Third, as a result of its single-centre scope the patient cohort is relatively small and therefore analysis of clinical meaningful subgroups limited. Fourth, the selected follow up period in this study might be too short to assess long term neurologic outcome, given the sometimes long reconvalescence after brain injury.

## Conclusions

A reduced GWR is associated with unfavourable neurologic outcomes in comatose patients after resuscitation of cardiac arrest. Sensitivity of GWR for predicting neurologic outcome seems limited within the first 12 h after cardiac arrest, pointing to a time window of 12 to 48 h after ROSC with a reasonable trade-off between diagnostic accuracy and the demand of the treating clinicians for early outcome prediction. We propose a threshold for GWR at caudate nucleus and internal capsule of 1.17. This threshold needs to be validated in a larger trial powered for confirming its prognostic value. In particular, it has to be demonstrated that GWR maintains its prognostic power after multivariate analysis of potentially confounding conditions and comorbidities. The design of this trial needs to exclude an interaction between calculated GWR and fundamental treatment decisions, especially premature withdrawal of life sustaining therapies. Additionally, further studies are needed to reliably exclude an impact of therapeutic hypothermia on GWR diagnostic power or specific thresholds.

## Data Availability

The datasets generated and analysed during the current study are not publicly available due to privacy restrictions but are available from the corresponding author on reasonable request.

## Abbreviations

CPC: Cerebral performance category
CPR: Cardio-pulmonary resuscitation
CT: Computed tomography
GWR: Gray-white-matter-ratio
ICU: Intensive care unit
NSE: Neuron-specific enolase
MRI: Magnetic resonance imaging
OHCA: Out-of-hospital cardiac arrest
ROI: Region of interest
ROSC: Return of spontaneous circulation
SSEP: Somatosensory evoked potentials
WLST: Withdrawal of life-sustaining therapies
